# Tooth loss and adiposity: possible role of carnitine transporter (OCTN1/2) polymorphisms in women but not in men

**DOI:** 10.1007/s00784-020-03594-w

**Published:** 2020-09-22

**Authors:** Peter Meisel, Stefanie Pagels, Markus Grube, Gabriele Jedlitschky, Henry Völzke, Thomas Kocher

**Affiliations:** 1 Department of Periodontology, Dental Clinics, Dental School, University Medicine Greifswald, Fleischmannstrasse 42, 17475 Greifswald, Germany; 2 Department of Pharmacology of the Center of Drug Absorption and Transport (C_DAT), University Medicine Greifswald, Greifswald, Germany; 3 Institute for Community Medicine, University Medicine Greifswald, Greifswald, Germany

**Keywords:** Tooth loss, OCTN1/2, Polymorphism, Obesity, Sex, Carnitine

## Abstract

**Objective:**

*SLC22A4/5* single nucleotide polymorphisms (SNPs) have been reported to affect inflammatory diseases. We report the relationship of these polymorphisms with adiposity and tooth loss as elucidated in a 10-year follow-up study.

**Methods:**

Participants of the Study of Health in Pomerania (SHIP, *N* = 4105) were genotyped for the polymorphisms c.1507C > T in *SLC22A4* (rs1050152) and -207C > G in *SLC22A5* (rs2631367) using allele-specific real-time PCR assays. A total of 1817 subjects, 934 female and 883 male aged 30–80 years, underwent follow-up 10 years later (SHIP-2) and were assessed for adiposity and tooth loss.

**Results:**

The frequencies of the rarer *SLC22A4* TT and *SLC22A5* CC alleles were 16.7% and 20.3%, respectively. In women, tooth loss was associated with genotype TT *vs.* CC with incidence rate ratio IRR = 0.74 (95% C.I. 0.60–0.92) and CC *vs.* GG IRR = 0.79 (0.65–0.96) for *SLC22A4* and *SLC22A5* SNPs, respectively. In men, no such associations were observed. In the follow-up examination, the relationship between tooth loss and these SNPs was in parallel with measures of body shape such as BMI, body weight, waist circumference, or body fat accumulation. The association between muscle strength and body fat mass was modified by the genotypes studied.

**Conclusions:**

*SLC22A4* c.150C > T and *SLC22A5* -207C > G polymorphisms are associated with tooth loss and markers of body shape in women but not in men.

**Clinical relevance:**

Tooth loss may be related to obesity beyond inflammatory mechanisms, conceivably with a genetic background.

**Electronic supplementary material:**

The online version of this article (10.1007/s00784-020-03594-w) contains supplementary material, which is available to authorized users.

## Introduction

In our functional studies on human membrane transporter proteins [1], we detected the expression of the organic cation transporters novel (OCTNs) in human gingival tissues by histochemical means. Taking this expression as a prerequisite, we observed a polymorphic gene-dose effect of the OCTNs on adiposity and tooth loss in a general population study. Various studies reported associations between obesity and tooth loss but a common genetic background is not known [2–4]. Though still unexplained, in this report we want to describe the possible associations.

The OCTNs encoded by the *SLC22* gene family mediate transport processes of diverse organic electrolytes, that is, molecules that are generally charged. The OCTNs support transport of selected zwitterions such as carnitine, acetylcholine, or ergothioneine. They are physiologically important for maintaining systemic and tissue levels of carnitine by regulating its intestinal absorption, tissue distribution, and renal reabsorption. Functional polymorphisms of the genes encoding these transporters were detected in *SLC22A4* (c.1672C > T; rs1050152) for OCTN1 as a missense mutation leading to an amino acid substitution L503F and in *SLC22A5* (rs2631367) for OCTN2 as a transversion in the promoter region leading to a reduced promotor activity. It has been shown that the single nucleotide polymorphisms (SNPs) of these genes encoding the transporters are associated with diseases such as Crohn’s disease, psoriasis, diabetes, and rheumatoid arthritis [5]. The association of the polymorphism with diseases was first reported 2004 by Peltekova et al. [6]^.^ A defect of OCTN2 due to genetic loss of function variants causes systemic primary carnitine deficiency associated with muscle weakness and fatty liver disease and other pathologies [7].

Common risk factors together with an inflammatory background suggest that these pathologies may be associated with periodontitis. Periodontitis is associated with inflammatory bowel diseases [8]. Likewise, associations with periodontitis are also evident with psoriasis [9], diabetes [10], and rheumatoid arthritis [11]. There is evidence that Crohn’s disease, rheumatoid arthritis, and psoriasis have common genetic backgrounds including the OCTN1/2 transporters [12].

Besides trauma and orthodontic treatment, caries and periodontitis are the main causes of tooth loss in the general population. Both of them have a strong genetic background [13]. In recent years, an increasing interest has addressed the association between periodontal diseases and other inflammatory diseases such as cardiovascular diseases, diabetes, rheumatoid arthritis, and obesity. It is well established that periodontitis is associated with adiposity parameters such as body mass index (BMI), waist-to-hip ration (WHR), or waist circumference (WC) among others [14]. In the past, many efforts were also made to elucidate possible associations in the pathogenesis between Crohn’s disease and periodontal diseases [15, 16]. Both Crohn’s disease and periodontitis are chronic inflammatory diseases characterized by microbial challenges, an exaggerating inflammatory response, and sharing some risk factors. Such common risk factors are obesity, smoking, diet, and, probably, heritability modified by environmental factors. Associations between caries and Crohn’s disease suggest that, despite inconclusive, tooth loss may be a matter of some concern in this bowel disease [17]. A bi-directional relationship may exist between the local periodontitis and systemic diseases, both of which are inflammatory in nature. Though speculative, such an association is conceivable if it is true that pathological states such as Crohn’s disease, diabetes, psoriasis, and rheumatoid arthritis, all of them associated with OCTN polymorphisms, are also connected or related to periodontitis. We can hypothesize that also tooth loss as the most prominent consequence of periodontitis could be associated with these polymorphisms, i.e., the SNPs of OCTN1 and/or OCTN2. Therefore, it was the aim of the present study to assess whether there are associations of the polymorphic genetic variants of these transporters with adiposity and tooth loss in a large population-based cohort. Special attention was given to the observed sex-related differences**.**

## Material and methods

### Study population

The Study of Health in Pomerania (SHIP) is a longitudinal population-based health study, which started from a 20- to 81-year-old population in Northeastern Germany. SHIP has the objectives to assess prevalence and incidence of common risk factors, subclinical disorders, and clinical diseases and to investigate the complex associations among these. Baseline study SHIP-0 was based on a representative sample examined from 1997 to 2001 in West Pomerania including 4308 participants [18]. Ten years later, 2333 subjects were re-examined in SHIP-2. We excluded participants younger than 30 years and those with edentulism at baseline. With complete genotyping, 1817 participants were included, 934 and 883 female and male, respectively. Different figures due to failed genotyping will be indicated. All participants gave their written informed consent. The study was approved by the local ethics committee. The STROBE guidelines were followed in the reporting of this observational study.

### Dental variables

The periodontal status was recorded according to the half-mouth method on the right or left side in alternating subjects. The manual periodontal probe PCP11 (Hu-Friedy, Chicago, IL, USA) was used. Probing depth (PD) is the distance from the gingival margin to the bottom of the periodontal pocket. Clinical attachment level (CAL) is represented by the distance from the cemento-enamel junction to the bottom of the periodontal pocket. Measurements were assessed at four sites per tooth (mesiobuccal, midbuccal, distobuccal, and midlingual/midpalatinal) and recorded as whole millimeters. The complete number of teeth excluding third molars was counted (maximum 28). Caries data were dichotomized according to the presence or absence of any decayed sites. Baseline data were assessed from questionnaires: frequency of dental visits over the last year, time of last dental visit, reporting mobile teeth (yes/no), use of mobile dentures, income, smoking (3 categories never, quitted, current), and education (3 categories according to the final school grade). Tooth loss was categorized as dichotomized loss of any tooth lost during the follow-up period (yes/no) or the number of teeth lost counted.

### Laboratory and anthropometric data

DNA was extracted from venous blood samples and the genotype of each individual determined. The polymorphisms c.1507C > T in *SLC22A4* and -207C > G in *SLC22A5* were assessed using allele-specific real-time PCR assays. We used the predeveloped TaqMan SNP Genotyping Assays C___3170459_30 (*SLC22A4*; rs1050152) and C__26479161_30 (*SLC22A5*; rs2631367 assay kits purchased from Life Technology, Thermo Fisher Scientific (Darmstadt, Germany). Genotyping was performed on a QuantStudio™ 12 K Flex Real-Time PCR System with 96 well fast block using a TaqMan genotyping mastermix according to the manufacturer’s instruction.

A non-fasting blood sample was drawn from the antecubital vein in the supine position and immediately analyzed or stored at − 80 °C, fibrinogen and glycated hemoglobin (HbA1c) with standard laboratory methods. High-sensitivity CRP was determined in serum by particle-enhanced immuno-nephelometry (hsCRP kit, Dade Behring Inc.); test sensitivity was 0.2 mg/L.

Anthropometric measurements were taken under standardized conditions using balance and height measuring devices (SOEHNLE, Murrhardt, Germany). Body weight was measured to the nearest 0.10 kg on a decimal scale, height to the nearest 1 cm, and waist and hip circumferences to the nearest 0.5 cm. Waist girth was measured at the midpoint between the lower ribs and the iliac crest. Hip circumference was measured horizontally at the level of the largest lateral extension of the hips or over the buttocks. Hand grip strength was used as a proxy for the general health state of the participants strongly associated with obesity [19]. Hand grip strength was measured by handheld Smedley-type dynamometer used for diagnostic purposes (Scandidact, Denmark); it was indicated in kilograms. We measured grip strength left- and right-handed and used the maximum strength of either hand as an independent variable.

Lean body mass, body fat mass, and basal metabolic rate were derived from bioelectrical impedance analysis (BIA) using a multifrequency Nutriguard-M device (Data Input, Pöcking, Germany). The electrodes were placed on hand, wrist, ankle, and foot and test frequencies were measured at 5, 50, and 100 kHz according to the manufacturer’s instructions [20].

### Statistics

Baseline data were employed only from those participants still present in the follow-up examinations. Means and standard deviations (SD) were computed for continuous variables, whereas frequency distributions were assessed in contingency tables for categorical variables. Kruskal-Wallis tests or contingence tables were used to assess differences in continuous and categorical variables, respectively. Incidence risk rates of future tooth loss were derived and related to the genetic polymorphisms assessed. Follow-up time varied between 9.2 and 14.5 years, median 10.4 years. Negative binomial regressions were employed to model tooth loss with impact of *SLC22A4/5* genotypes and anthropomorphic measures. For sensitivity analyses, we repeated the regressions with subjects aged 40–70 years and having five or more teeth at baseline (*N* = 983; i.e., 834 were sorted out). Software STATA 14.2 (College Station, TX) was used. The analysis program HapView (Broad Institute, Cambridge, MA) was used to analyze haplotypes and linkage disequilibrium. Statistical significance was set at *p* < 0.05, for interaction at *p* < 0.10.

## Results

### Baseline characteristics

The data relevant to tooth loss including adiposity are compiled in Table 1 including the distribution of allelic variants of the *SLC22A4* gene. Relevant risk factors for tooth loss were different between women and men, especially the dental and anthropometric measures, but also laboratory and behavioral factors were quite different in men as compared with women. Sex-specific differences in socio-economic status may be important. An exception was the concentration of CRP as well as fibrinogen, which was higher in women. This indicates profound differences in all of these risk factors; however, the mean number of teeth was not different between the sexes. The frequencies of the rarer *SLC22A4* T variant allele and the rarer *SLC22A5* C variant in this population were 40.8% and 44.9%, respectively. Frequency of T alleles of *SLC22A4* and that of C alleles of *SLC22A5* was slightly reduced in men, albeit an insignificant difference. Hardy-Weinberg equilibrium was fulfilled in both women and men. *SLC22A4* and *SLC22A5* exist in strong linkage disequilibrium (D’ 0.99, *r*^2^ = 0.83); the homozygous TC haplotype with a frequency of 16.4% (95% confidence interval 14.5–18.4%).

**Table 1:** Characteristics of participants according to sex at baseline

	Women (N = 934)	Men (N = 883)	*p*
Age at baseline, years	48.7 ± 11.3	49.9 ± 11.7	0.030
BMI, kg/m^2^	26.5 ± 4.8	27.7 ± 3.8	< 0.001
Waist-to-hip ratio, WHR	0.80 ± 0.06	0.93 ± 0.06	< 0.001
No. of teeth, median (IQR) *	22 (18–25)	23 (18–26)	0.023
Mean CAL, mm	2.4 ± 1.5	2.9 ± 1.8	< 0.001
Mean PD, mm	2.4 ± 0.6	2.6 ± 0.7	< 0.001
% plaque, median (IQR)	45 (21–71)	54 (30–81)	< 0.001
No. reported mobile teeth (%)	99 (10.6)	117 (13.3)	0.081
Caries, no. with decayed sites (%)	179 (19.2)	178 (20.2)	0.73
No. of last dental visit > 1 year (%)	55 (5.9)	83 (9.4)	0.005
Income per month, 1000$	2.17 ± 1.06	2.38 ± 1.14	< 0.001
No. of current smokers (%)	217 (23.3)	343 (38.8)	< 0.001
No. of educated < 10th grade (%)	254 (27.2)	272 (30.8)	0.090
Glycated hemoglobin HbA1c, %	5.2 ± 0.7	5.5 ± 0.8	< 0.001
Fibrinogen, g/L	3.0 ± 0.6	2.9 ± 0.6	< 0.001
Median CRP, mg/L (IQR)	1.3 (0.6–3.2)	1.1 (0.6–2.2)	0.002
*SLC22A4*—CC, count (%)	308 (33.0)	323 (36.6)	
*SLC22A4*—CT	459 (49.1)	416 (47.1)	0.25
*SLC22A4*—TT	167 (17.9)	144 (16.3)	

Data are presented as number (percentage) or mean ± SD or plaque as median (*IQR* interquartile range)

*Edentulous participants excluded

### Follow-up observations

**Fig. 1:**
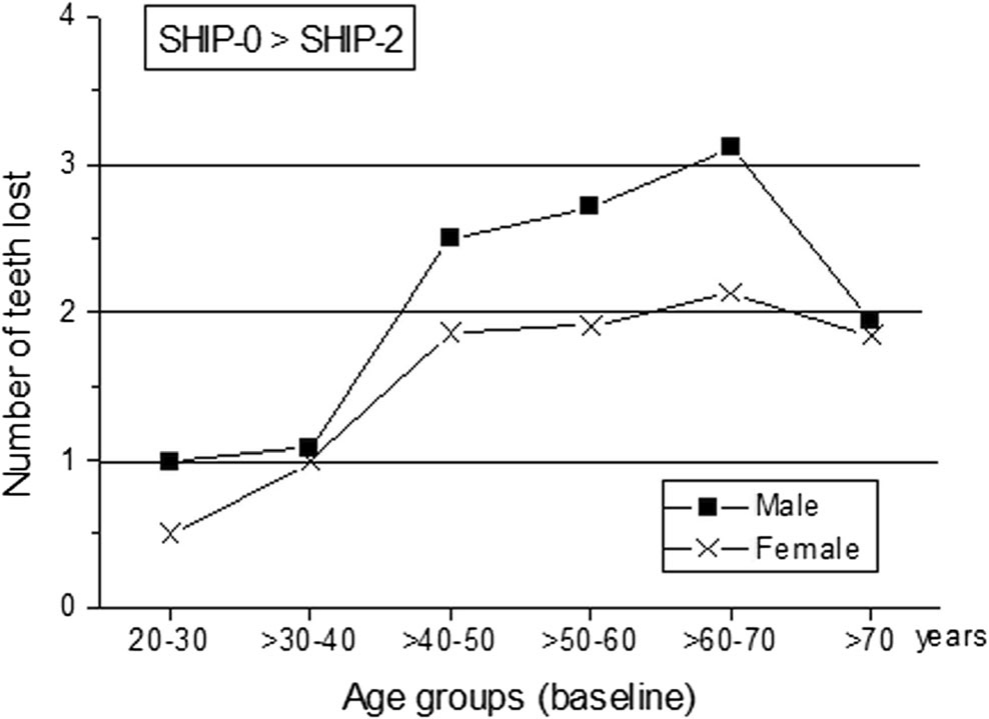
Tooth loss in women and men during the 10-year follow-up period in six age cohorts

Differences in the number of teeth lost between women and men during the follow-up time are shown in Fig. 1 for different age groups. The most striking differences in tooth loss between the sexes were observed at ages 40–70 years. In this age group, men lost on average 0.6–1.0 teeth more than women within the follow-up period. The contrary difference was observed with weight gain across the age groups. BMI increases were higher in women than in men in all age groups assessed.

In Table 2, we show the initially found association of BMI and obesity frequencies with the *SLC22A4* genotypes revealing gene dose effects among the baseline as well as in the follow-up participants. These associations were observed in female but not in male participants.

**Table 2:** Distribution of BMI and obese participants across the *SLC22A4* genotype2 stratified by sex: female (A) or male (B)

A	Female participants	CC (*N* = 308)	CT (*N* = 459)	TT (*N* = 167)
BMI, kg/m^2^ at baseline		27.0 ± 5.0	26.4 ± 4.8	26.0 ± 4.7
BMI, kg/m^2^ at follow-up time		28.7 ± 5.3	27.8 ± 5.2	27.6 ± 5.1
No. with obesity (%) at baseline		73 (23.7)	103(22.4)	32 (19.2)
No. with obesity (%) at follow-up		113 (36.7)	133 (29.0)	46 (27.5)
B	Male participants	CC (*N* = 323)	CT (*N* = 416)	TT (*N* = 144)
BMI, kg/m^2^ at baseline		27.7 ± 3.7	27.8 ± 3.9	27.6 ± 3.6
BMI, kg/m^2^ at follow-up time		28.9 ± 4.0	28.8 ± 4.2	28.9 ± 4.1
No. with obesity (%) at baseline		84 (26.0)	101 (24.3)	29 (20.1)
No. with obesity (%) at follow-up		128 (39.6)	138 (33.2)	50 (34.7)

Table 3 summarizes the distribution of genotypes for *SLC22A4* and *SLC22A5* among the participants experiencing no tooth loss at all and those with tooth loss during the follow-up period. In women, tooth loss was associated with lower frequencies of TT homozygotes and CC homozygotes of *SLC22A4* and *SLC22A5*, respectively. Thus, reduced incidence rate ratios of tooth loss were estimated. In men, such distributional differences were not observed, neither in *SLC22A4* nor in *SLC22A5* genotype distributions. Accordingly, in the supplement Table S1, the corresponding figures for the haplotypes TC are collected. The association between tooth loss and *SLC22A4* configuration of women was also stratified by menopause status revealing no difference between premenopausal and postmenopausal women (not shown).

**Table 3:** Distribution of genotype constellations according to tooth loss in female or male participants

*SLC22A4* Women	With tooth loss (*N* = 505)	Without tooth loss (*N* = 429)	IRR* (95% C.I.)	*p*
CC (%)	176 (34.9)	132 (30.8)	1	Ref.
CT (%)	254 (50.3)	205 (47.8)	0.97 (0.85–1.10)	0.62
TT (%)	75 (14.9)	92 (21.5)	0.79 (0.65–0.95)	0.011
*SLC22A4* Men	With tooth loss (*N* = 516)	Without tooth loss (*N* = 367)	IRR* (95% C.I.)	*p*
CC (%)	192 (37.2)	131 (35.7)	1	Ref.
CT (%)	237 (45.9)	179 (48.8)	0.96 (0.85–1.08)	0.50
TT (%)	87 (16.9)	57 (15.5)	1.01 (0.87–1.19)	0.84
*SLC22A5* Women	With tooth loss (*N* = 495)	Without tooth loss (*N* = 421)	IRR* (95% C.I.)	*p*
GG (%)	145 (29.3)	109 (25.9)	1	Ref.
CG (%)	259 (52.3)	206 (48.9)	0.97 (0.85–1.10)	0.72
CC (%)	91 (18.4)	106 (25.2)	0.79 (0.65–0.95)	0.022
*SLC22A5* Men	With tooth loss (*N* = 505)	Without tooth loss (*N* = 360)	IRR* (95% C.I.)	*p*
GG (%)	166 (32.9)	103 (28.6)	1	Ref.
CG (%)	237 (46.9)	188 (52.2)	0.90 (0.80–1.03)	0.12
CC (%)	102 (20.2)	69 (19.2)	0.97 (0.83–1.13)	0.67

*IRR incidence rate ratio

The regression modelling of tooth loss during 10 years adjusted to the main risk factors affirms the sex differences by the genotypes and, additionally, modified by the distinct influence of anthropomorphic measures such as obesity (Table 4). Again, in women, tooth loss was associated with lower frequencies of TT homozygotes and CC homozygotes of *SLC22A4* and *SLC22A5*, respectively, Table 5.

**Table 4:** Tooth loss during 10 years regressed on baseline factors and configuration of *SLC22A4 or SLC22A5* genotypes in women and men, incidence rate ratios of tooth loss (IRR, 95% confidence intervals)

*SLC22A4*		Women IRR (95% CI)	*p*	Men IRR (95% CI)	*p*
Mean PD, mm		2.35 (1.98–2.80)	< 0.001	2.25 (1.95–2.61)	< 0.001
Caries, fraction (0-1)		1.35 (1.07–1.72)	0.012	1.32 (1.07–1.63)	0.011
Mobile teeth reported		1.72 (1.30–2.28)	< 0.001	1.76 (1.38–2.24)	< 0.001
Frequency of dental visits/year		1.01 (0.98–1.04)	0.60	1.04 (1.01–1.08)	0.015
Interaction *SLC22A4* TT × Obesity					
0	0	1	Ref.	1	Ref.
0	1	0.98 (0.77–1.25)	0.87	1.23 (0.99–1.53)	0.062
1	0	0.70 (0.52–0.93)	0.014	0.98 (0.74–1.29)	0.88
1	1	0.48 (0.27–0.88)	0.017	1.17 (0.71–1.93)	0.54
*SLC22A5*		IRR (95% CI)	*p*	IRR (95% CI)	*p*
Mean PD, mm		2.35 (1.98–2.80)	< 0.001	2.25 (1.94–2.60)	< 0.001
Caries, fraction (0-1)		1.36 (1.07–1.72)	0.012	1.32 (1.07–1.64)	0.010
Mobile teeth reported		1.71 (1.29–2.26)	< 0.001	1.76 (1.38–2.24)	< 0.001
Frequency of dental visits/year		1.01 (0.98–1.04)	0.60	1.04 (1.01–1.08)	0.015
Interaction *SLC22A5* CC × Obesity					
0	0	1	Ref.	1	Ref.
0	1	0.98 (0.76–1.25)	0.87	1.26 (1.01–1.58)	0.040
1	0	0.71 (0.54–0.93)	0.013	1.03 (0.79–1.33)	0.84
1	1	0.54 (0.32–0.91)	0.020	1.10 (0.71–1.70)	0.68

*Additionally adjusted for age, number of teeth at baseline, HbA1c, education (3 categories), income (1000$), smoking (3 categories)

**Table 5:** Outcome characteristics of the follow-up study participants by their *SLC22A4* genotype and stratified by sex: female (A) or male (B)

A	Female participants	CC (*N* = 308)	CT (*N* = 459)	TT (*N* = 167)	*p*
Age at follow-up time		58.5 ± 10.9	59.4 ± 11.4	60.1 ± 11.1	0.36
Mean number of teeth		18.6 ± 7.6	18.8 ± 7.7	18.2 ± 8.3	0.86
Participants who lost any teeth (%)		176 (57.1)	254 (55.3)	75 (44.9)	0.029
Participants who lost 1–2 teeth (%)		108 (35.1)	147 (32.0)	42 (25.2)	0.033
No. of teeth lost during 10 years		1.6 ± 2.4	1.8 ± 2.9	1.3 ± 2.6	0.055
BMI, kg/m^2^		28.7 ± 5.3	27.8 ± 5.2	27.6 ± 5.1	0.018
Lean body mass, kg		48.9 ± 5.6	47.0 ± 5.2	47.1 ± 5.3	< 0.001
Body fat mass, kg		27.7 ± 10.0	25.4 ± 9.2	25.5 ± 9.2	0.006
Basal metabolic rate, kcal		1384 ± 118	1353 ± 101	1351 ± 112	< 0.001
Hand grip strength, normalized *		0.98 ± 0.29	0.97 ± 0.31	1.02 ± 0.26	0.14
B	Male participants	CC (*N* = 323)	CT (*N* = 416)	TT (*N* = 144)	*p*
Age at follow-up time		60.2 ± 11.6	60.6 ± 12.0	60.7 ± 9.9	0.76
Mean number of teeth		18.7 ± 8.1	18.7 ± 8.4	18.3 ± 8.4	0.81
Participants who lost teeth (%)		(59.4)	(57.0)	(60.4)	0.99
Participants who lost 1–2 teeth (%)		109 (33.8)	123 (29.6)	47 (32.6)	0.50
No. of teeth lost during 10 years		2.2 ± 3.3	2.0 ± 3.0	2.4 ± 3.7	0.67
BMI, kg/m^2^		28.9 ± 4.0	28.8 ± 4.2	28.9 ± 4.1	0.67
Lean body mass, kg		66.6 ± 8.6	66.5 ± 8.8	66.6 ± 8.7	0.96
Body fat mass, kg		21.9 ± 7.3	21.9 ± 7.7	22.0 ± 6.8	0.78
Basal metabolic rate, kcal		1722 ± 179	1712 ± 174	1718 ± 168	0.84
Hand grip strength, normalized *		1.58 ± 0.37	1.58 ± 0.37	1.61 ± 0.36	0.73

*kg/BMI

We compared the outcome figures of tooth loss with indices of body shape and adiposity in the follow-up data in relation to the three genotypes each of *SCL22A4* (Table 4) for women and men separately. Again, an association of tooth loss with the genotypes studied was only present in women but not in men. Neither for periodontal measures CAL nor for PD such a relationship was identifiable (not shown). Besides tooth loss, most obvious was the decrease of all body shape–related parameters across the *SLC22A4* genotypes from CC to CT to TT. The association between genetic configurations with tooth loss was parallel to the association with the body shape parameters notwithstanding the fact that there was an increase in BMI in both women and men from baseline to follow-up. There was a consistent trend of all adiposity figures across the genotypes coincident with tooth loss. However, whereas reduced tooth loss was associated only with the homozygous variants TT or CC, anthropometric measures were reduced also with the heterozygous genotypes. Higher percentage of body fat mass in women as compared with men was obvious. Markers of inflammation exhibited an analogous tendency, without reaching significance. The corresponding results regarding the *SLC22A5* genotypes can be found in the supplement designated as Table S2.

When adjusted to BMI, hand grip strength was highly correlated to body fat mass especially in female participants (not shown). This association was not different across the genotypes, neither for *SLC22A4* nor for *SLC22A5* SNPs.

### Sensitivity analyses

The above reported analyses included all participants who were older than 30 years and were not edentulous at baseline. The genotype distributions analyses given in Table 3 were repeated after all participants with less than six teeth were excluded and, moreover, only participants aged 40–70 years were included (*N* = 983 remained, 494 female and 489 male). In this case, the remaining participants represent the group characterized by the most marked sex-related differences in tooth loss, as illustrated in Fig. 1. Table S3 (supplement) gives the results completely in accordance with those of the total study group. All these maneuvers did not change the association between *SLC22A4* SNPs, obesity, and tooth loss. Likewise, adiposity measures and tooth loss distributed across the remaining TC haplotypes were in accordance with the complete data set (supplement Table S4).

## Discussion

Obesity and tooth loss are both of major health concern. Tooth loss affects mastication, well-being, and quality of life [21]. Moreover, tooth loss is associated with impaired systemic health including obesity, morbidity, and even mortality [22–24]. Besides numerous environmental risks, tooth loss is also substantially determined by genetic factors [25]. Both the environmental and biological factors for tooth loss are differentially affected by sex and/or gender with characteristic differences in body shape [26]. Among them, the relationships to obesity are poorly understood.

In the present study, we observed that:

- Female homozygous TT carriers of *SLC22A4* had less tooth loss as compared with carriers of the C allele. Homozygous carriers CC of *SLC22A5* were found with lower tooth loss rates than subjects with the G alleles, also only in women.
- Incidence risk rates of tooth loss were substantially decreased in carriers of these gene constellations being in linkage disequilibrium. Accordingly, this is also true for the haplotype constellation TC.
- Parallel to these observations, the T allele of *SLC22A4* was associated with decreased adiposity indicated by BMI, abdominal girth, and body fat as compared with the C carriers in women but not in men. The C allele of *SLC22A5* was also associated with decreased adiposity, likewise only in women but not in men.
- Periodontitis or markers of inflammation as cause of tooth loss did not attenuate these relationships. Hand grip strength, probably related to carnitine metabolism, was not itself related to the SNPs despite a strong association between muscle strength and BMI or body fat mass and also between muscle strength and number of teeth. Our initial hypothesis is to be rejected that a common inflammatory mechanism could be the link between periodontitis and those diseases which are themselves associated with the OCTN polymorphisms.

Associations of these polymorphisms with dental conditions have never been described before. To our knowledge, the polymorphisms were also not reported in GWAS data with respect to caries, periodontitis or tooth loss.

The extent of genetic effects on adiposity was described as comparable or even equal in both sexes [27].

For now, any mechanistic explanations are speculative; however, some noteworthy relationships to other conditions are obvious. Diseases associated with the *SLC22A4/5* polymorphisms were mentioned in the introduction. Crohn’s disease is associated with the T alleles in *SLC22A4* in contrast to our observation of reduced tooth loss with the TT constellation [28, 29]. In psoriatic arthritis and Crohn’s disease GG; homozygotes of *SLC22A5* were under-represented in affected subjects compared to controls [12, 28]. Sex differences were not reported. Associations of Crohn’s disease with periodontitis, caries, and other risk factors for tooth loss are well known [16, 30, 31]. Tooth loss, however, was scarcely reported. Yin et al. [32] reported in a huge population-based study in Sweden that tooth loss was associated with a lower risk of inflammatory bowel diseases with a hazard ratio of 0.56. This is a substantial, albeit circumstantial, evidence to suggest an unknown interrelationship. Taking into account that Crohn’s disease is associated with those SNPs which in our study show diminished tooth loss, this finding is in line with our results. Certainly, tooth loss is rather a problem if ulcerative colitis, less so of Crohn’s disease [33].

The OCTN1/2 carnitine transporters and their polymorphisms probably affect the disposition of carnitine and its congeners. The carnitine metabolism is important for muscle metabolism and body shape and is correlated to obesity [34]. Important sex differences exist in carnitine disposition as in body shape characteristics [35, 36]. Correspondingly, muscle strength is correlated to carnitine levels in women but not in men [37]. Obesity, muscle strength, and masticatory ability are closely related to the number of retained teeth [38]. On the other hand, obesity is a risk factor for tooth loss via periodontitis mediated by inflammation. This is particularly relevant in men, less so in women [22]. In the present study, periodontitis measures such as PD or CAL or markers of inflammation were not associated with OCTN1/2 genotype configurations but anthropomorphic measures were related in women but not in men.

Obesity is a risk factor for periodontitis and tooth loss and is also strongly connected with Crohn’s disease [39, 40]. Carnitine transport and metabolism is affected by the SNPs mentioned above. Plasma carnitine and muscle strength are different between women and men [41]. In the present study, muscle strength was closely related to obesity but not to genotypes related to diminished tooth loss. However, carnitine disposition modified by the OCTN transporters may be related to muscle strength with sex differences [7, 37]. Also, an association between periodontitis and tooth loss with muscle strength (measured by hand grip strength) was reported [42]. Sex differences were described in all these studies leading to the conclusion that body shape differences are important [43]. Moreover, hormonal influences are to taken into account as there is a reason to presume that hormones interfere with carnitine metabolism und transport [41, 44]. We found differences between pre- and postmenopausal women in the association with muscle strength but not with tooth loss. Sex differences in body fat distribution usually characterized as gynoid or android obesity are determinant for systemic diseases and encompass inherited and metabolic impact on health including tooth retention or loss [45, 46]. With respect to sex differences, it is known that extra-intestinal manifestations of inflammatory bowel diseases are more prevalent in female patients than in males [47].

Under the impression that there are doubts about these unexplainable results, we analyzed different subgroups, such as different age groups, two polymorphisms, different baseline teeth, differentiating between extent of tooth loss, pre- and postmenopausal women. In every case, identical results were achieved in female participants. Nevertheless, some open questions remain to be clarified. In the genetic relationships to tooth loss shown, we found no connection to periodontal parameters such as attachment loss or probing depth. Residual confounding may be assumed. Still the biological basis remains vague, even though the possibility of an unknown genetic trait in women is conceivable.

In this study, it was not possible to draw a causal connection between the reported tooth loss and the OCTN1/2 polymorphisms. This was a general population study without selection for any disease state, let alone for intestinal bowel diseases. Hand grip strength and fat and impedance analyses of fat distribution were not available in the baseline data but only in the follow-up. Nevertheless, there are some parallels between peculiarities of tooth loss related to adiposity with those diseases associated with the SNPs studied, and the sex differences underlying all of them. The study needs replication in independent populations before reliable conclusions should be drawn. Such sex-specific data could allow clinicians to better tailor prophylaxis or care to individuals.

## Electronic supplementary material

ESM 1 (DOCX 30 kb).
