# Correction to: Tooth loss and adiposity: possible role of carnitine transporter (OCTN1/2) polymorphisms in women but not in men

**DOI:** 10.1007/s00784-021-04025-0

**Published:** 2021-06-28

**Authors:** Peter Meisel, Stefanie Pagels, Markus Grube, Gabriele Jedlitschky, Henry Völzke, Thomas Kocher

**Affiliations:** 1 Department of Periodontology, Dental Clinics, Dental School, University Medicine Greifswald, Fleischmannstrasse 42, 17475 Greifswald, Germany; 2 Department of Pharmacology, Center of Drug Absorption and Transport (C_DAT), University Medicine Greifswald, Greifswald, Germany; 3 Institute for Community Medicine, University Medicine Greifswald, Greifswald, Germany

## Abstract

The online version of the original article can be found at https://doi.org/10.1007/s00784-020–03594-w

**Correction to** **:** **Clinical Oral Investigations**

https://doi.org/10.1007/s00784-020–03594-w

The article “Tooth loss and adiposity: possible role of carnitine transporter (OCTN1/2) polymorphisms in women but not in men”, written by Peter Meisel, Stefanie Pagels, Markus Grube, Gabriele Jedlitschky, Henry Völzke and Thomas Kocher, was originally published Online First without Open Access. After publication in volume 25, issue 2, page 701–709 the author decided to opt for Open Choice and to make the article an Open Access publication. Therefore, the copyright of the article has been changed to © The Author(s) 2021 and the article is forthwith distributed under the terms of the Creative Commons Attribution 4.0 International License, which permits use, sharing, adaptation, distribution and reproduction in any medium or format, as long as you give appropriate credit to the original author(s) and the source, provide a link to the Creative Commons license, and indicate if changes were made. The images or other third party material in this article are included in the article’s Creative Commons license, unless indicated otherwise in a credit line to the material. If material is not included in the article’s Creative Commons license and your intended use is not permitted by statutory regulation or exceeds the permitted use, you will need to obtain permission directly from the copyright holder. To view a copy of this license, visit http://creativecommons.org/licenses/by/4.0.

The original article has been corrected.

