# Supplementary material for: Tooth loss and adiposity: possible role of carnitine transporter (OCTN1/2) polymorphisms in women but not in men
Source: Clin Oral Investig. 2020 Sep 22;25(2):701–9. doi: 10.1007/s00784-020-03594-w (PMC8208909; doi:10.1007/s00784-020-03594-w)
Supplement: Supplementary file 1 — (DOCX 30 kb). [file 784_2020_3594_MOESM1_ESM.docx]

**Supplement**

**Tooth loss and adiposity: Possible role of carnitine transporters (OCTN1/2) polymorphisms in women but not in men**

Peter Meisel ^1^, Stefanie Pagels ^1,2^, Markus Grube ^2^, Gabriele Jedlitschky ^2^, Henry Völzke ^3^, Thomas Kocher ^1^

^1^ Dental School, Dental Clinics, Department of Periodontology, University Medicine Greifswald, Greifswald, Germany

^2^ Department of Pharmacology of the Center of Drug Absorption and Transport (C_DAT), University Medicine Greifswald, Greifswald, Germany

^3^ Institute for Community Medicine, University Medicine Greifswald, Greifswald, Germany

**Table S1.** Distribution of haplotype TC constellations by tooth loss in women (A) or men (B)

| **A Women** | **With tooth loss (N=409)** | **Without tooth loss (N=313)** | **IRR***  **(95% C.I.)** | **P** |
| --- | --- | --- | --- | --- |
| ***Haplotype TC*** |  |  |  |  |
| H0 (%) | 141 (34.5) | 98 (31.3) | 1 | ref. |
| heterozygous (%) | 214 (52.3) | 147 (47.0) | 1.00 (0.88-1.15) | 0.94 |
| homozygous (%) | 54 (13.2) | 68 (21.7) | 0.75 (0.60-0.94) | 0.008 |
| **B Men** | **With tooth loss (N=460)** | **Without tooth loss (N=323)** | **IRR***  **(95% C.I.)** | **P** |
| ***Haplotype TC*** |  |  |  |  |
| H0 (%) | 160 (37.0) | 104 (38.0) | 1 | ref. |
| heterozygous (%) | 203 (46.9) | 128 (46.7) | 1.01 (0.89-1.15) | 0.86 |
| homozygous (%) | 70 (16.2) | 42 (15.3) | 1.03 (0.87-1.23) | 0.73 |

* IRR – incidence risk ratio

**Table S2.** Outcome characteristics of the follow-up study participants by their ***SLC22A5*** genotype and

Stratified by sex: female (A) or male (B)

| **A Female participants** | **CC (N=197)** | **CG (N=465)** | **GG (N=254)** | **p** |
| --- | --- | --- | --- | --- |
| Age at follow-up time | 59.6 ± 10.9 | 59.3 ± 11.4 | 58.9 ± 11.0 | 0.82 |
| No. of teeth | 18.4 ± 8.2 | 19.0 ± 7.7 | 18.5 ± 7.4 | 0.45 |
| Participants who lost any teeth (%) | 91 (46.2) | 259 (55.7) | 145 (57.1) | 0.042 |
| Participants who lost 1-2 teeth (%) | 47 (23.9) | 159 (34.2) | 88 (34.7) | 0.013 |
| No. of teeth lost during 10 years | 1.5 ± 2.7 | 1.7 ± 2.7 | 1.6 ± 2.4 | 0.14 |
| BMI, kg/m² | 27.8 ± 5.1 | 27.8 ± 5.4 | 28.7 ± 5.3 | 0.028 |
| Lean body mass, kg | 47.4 ± 5.3 | 47.2 ± 5.3 | 48.8 ± 5.6 | 0.001 |
| Body fat mass, kg | 26.0 ± 9.4 | 25.4 ± 9.3 | 27.8 ± 10.0 | 0.008 |
| Basal metabolic rate, kcal | 1355 ± 109 | 1356 ± 106 | 1381 ± 118 | 0.018 |
| Hand grip strength, normalized * | 1.03 ± 0.26 | 0.99 ± 0.31 | 0.99 ± 28 | 0.16 |
| **B Male participants** | **CC (N=171)** | **CG (N=425)** | **GG (N=269)** | **p** |
| Age at follow-up time | 60.8 ± 10.3 | 59.9 ± 11.9 | 61.0 ± 11.8 | 0.31 |
| No. of teeth | 18.4 ± 8.4 | 18.8 ± 8.4 | 18.1 ± 8.3 | 0.32 |
| Participants who lost any teeth (%) | 100 (59.7) | 237 (55.8) | 166 (61.7) | 0.29 |
| Participants who lost 1-2 teeth (%) | 55 (32.5) | 124 (29.2) | 90 (33.5) | 0.29 |
| No. of teeth lost during 10 years | 2.3 ± 3.6 | 2.0 ± 3.0 | 2.4 ± 3.5 | 0.39 |
| BMI, kg/m² | 28.9 ± 4.1 | 28.7 ± 4.2 | 29.0 ± 4.1 | 0.36 |
| Lean body mass, kg | 66.6 ± 8.7 | 66.5 ± 8.8 | 66.9 ± 8.6 | 0.79 |
| Body fat mass, kg | 22.2 ± 6.7 | 21.9 ± 7.7 | 22.0 ± 7.5 | 0.67 |
| Basal metabolic rate, kcal | 1716 ± 172 | 1716 ± 174 | 1723 ± 182 | 0.94 |
| Hand grip strength, normalized * | 1.61 ± 0.36 | 1.62 ± 0.36 | 1.60 ± 0.36 | 0.77 |

* kg/BMI

**Table S3.** Tooth loss during ten years regressed on baseline factors and configuration of *SLC22A4 or SLC22A5* genotypes in women and men with six or more teeth at baseline and restricted to age 40-70 years, incidence rate ratios of tooth loss (IRR, 95% confidence intervals)

| ***SLC22A4*** | **Women**  **IRR (95% CI)** | **p** | **Men**  **IRR (95% CI)** | **p** |
| --- | --- | --- | --- | --- |
| Mean PD, mm | 2.17 (1.78-2.64) | <0.001 | 2.22 (1.90-2.59) | <0.001 |
| Caries, fraction (0-1) | 1.10 (0.84-1.44) | 0.50 | 1.35 (1.07-1.71) | 0.013 |
| Mobile teeth reported | 1.76 (1.31-2.36) | <0.001 | 1.72 (1.34-2.20) | <0.001 |
| Frequency of dental visits/year | 1.02 (0.98-1.06) | 0.25 | 1.02 (0.98-1.06) | 0.33 |
| **Interaction *SLC22A4* TT × Obesity** | |  |  |  |
| **0 0** | 1 | ref. | 1 | ref. |
| **0 1** | 0.93 (0.71-1.21) | 0.59 | 1.26 (1.00-1.59) | 0.048 |
| **1 0** | 0.75 (0.54-1.03) | 0.078 | 0.89 (0.66-1.20) | 0.44 |
| **1 1** | 0.45 (0.21-0.94) | 0.035 | 1.08 (0.66-1.77) | 0.75 |
| ***SLC22A5*** | **IRR (95% CI)** | p | **IRR (95% CI)** | p |
| Mean PD, mm | 2.25 (1.84-2.76) | <0.001 | 2.24 (1.90-2.62) | <0.001 |
| Caries, fraction (0-1) | 1.10 (0.84-1.45) | 0.48 | 1.35 (1.07-1.72) | 0.013 |
| Mobile teeth reported | 1.75 (1.30-2.36) | <0.001 | 1.75 (1.36-2.26) | <0.001 |
| Frequency of dental visits/year | 1.02 (0.98-1.06) | 0.25 | 1.02 (0.98-1.06) | 0.36 |
| **Interaction *SLC22A4* TT × Obesity** | |  |  |  |
| **0 0** | 1 | ref. | 1 | ref. |
| **0 1** | 0.95 (0.73-1.25) | 0.58 | 1.32 (1.04-1.67) | 0.024 |
| **1 0** | 0.81 (0.60-1.09) | 0.16 | 0.96 (0.73-1.28) | 0.80 |
| **1 1** | 0.58 (0.32-1.07) | 0.082 | 1.02 (0.65-1.60) | 0.93 |

* Additionally adjusted for age, number of teeth at baseline, HbA1c, education, smoking

**Table S4.** Tooth loss and adiposity parameters after ten years follow-up distributed between TC haplotypes

of *SLC22A4/SLC22A5* in women and men with six or more teeth at baseline and restricted to age 40-70 years

| **A Female participants** | **No TC**  **(N=161)** | **heterozygote (N=253)** | **homozygote TC**  **(N=80)** | **p** |
| --- | --- | --- | --- | --- |
| Age at follow-up time | 63.0 ± 7.5 | 63.9 ± 7.8 | 63.4 ± 7.9 | 0.48 |
| No. of teeth | 18.3 ± 6.7 | 17.8 ± 7.3 | 18.9 ± 7.2 | 0.43 |
| Participants who lost any teeth (%) | 98 (60.9) | 154 (60.9) | 37 (46.3) | 0.062 |
| Participants who lost 1-2 teeth (%) | 55 (34.2) | 82 (32.4) | 18 (22.5) | 0.052 |
| No. of teeth lost during 10 years | 1.8 ± 2.4 | 2.2 ± 3.1 | 1.4 ± 2.2 | 0.077 |
| BMI, kg/m² | 29.7 ± 5.6 | 28.9 ± 5.4 | 27.8 ± 5.7 | 0.017 |
| Lean body mass, kg | 49.0 ± 5.7 | 47.5 ± 5.5 | 47.1 ± 5.3 | 0.023 |
| Body fat mass, kg | 29.4 ± 10.3 | 27.2 ± 9.3 | 25.7 ± 10.1 | 0.016 |
| Basal metabolic rate, kcal | 1377 ± 115 | 1349 ± 105 | 1338 ± 105 | 0.035 |
| Hand grip strength, normalized * | 1.00 ± 0.28 | 0.99 ± 0.30 | 1.04 ± 0.25 | 0.11 |
| **B Male participants** | **No TC**  **(N=176)** | **heterozygote (N=223)** | **homozygote TC (N=90)** | **p** |
| Age at follow-up time | 64.3 ± 7.4 | 63.8 ± 8.2 | 63.8 ± 7.1 | 0.71 |
| No. of teeth | 17.3 ± 8.0 | 18.6 ± 8.0 | 17.7 ± 8.2 | 0.20 |
| Participants who lost any teeth (%) | 121 (68.8) | 144 (64.6) | 55 (61.1) | 0.43 |
| Participants who lost 1-2 teeth (%) | 56 (31.8) | 70 (31.4) | 24 (26.7) | 0.48 |
| No. of teeth lost during 10 years | 3.2 ± 4.1 | 2.5 ± 3.4 | 2.8 ± 4.3 | 0.30 |
| BMI, kg/m² | 29.4 ± 4.3 | 29.6 ± 4.5 | 29.5 ± 4.2 | 0.96 |
| Lean body mass, kg | 66.8 ± 8.9 | 67.0 ± 9.4 | 66.4 ± 8.4 | 0.98 |
| Body fat mass, kg | 22.5 ± 7.4 | 23.1 ± 8.0 | 22.9 ± 6.5 | 0.84 |
| Basal metabolic rate, kcal | 1713 ± 175 | 1706 ± 171 | 1702 ± 151 | 0.98 |
| Hand grip strength, normalized * | 1.61 ± 0.36 | 1.60 ± 0.37 | 1.62 ± 0.34 | 0.88 |

* kg/BMI
